# Comments on Hynes *et al*. Prevalence of Marijuana Use among University Students in Bolivia, Colombia, Ecuador and Peru. *Int. J. Environ. Res. Public Health* 2015, *12*, 5233–5240

**DOI:** 10.3390/ijerph120911718

**Published:** 2015-09-17

**Authors:** Maria Claudia Martinez-Novack, Maria Teresa Ortiz-Ortiz, Bruno Castañeda-Carbajal, German F. Alvarado

**Affiliations:** Facultad de Ciencias de la Salud, Escuela de Medicina, Universidad Peruana de Ciencias Aplicadas; Alameda San Marcos Avenue, block 2, Chorrillos. Lima 09, Lima, Peru

**Keywords:** Cannabis, Latin America, epidemiology, prevalence proportion, participation rate

## Abstract

We have read and analyzed the article entitled “Prevalence of marijuana use among university students in Bolivia, Colombia, Ecuador and Peru”. We propose some objective points which could enhance the internal validity of the study (*i.e.*, we suggest to report participation proportions).

We have read the article entitled “Prevalence of marijuana use among university students in Bolivia, Colombia, Ecuador and Peru” with great interest [1]. In fact, we consider it to be of high importance to have estimated the drug use among students of the Andean Community and its evolution over time. We also want to highlight the commendable effort to collect the data from these four Latin American countries. This study could be important to improve public health initiatives at the level of individual countries and also within the Andean Community.

There have been previous studies concerning the evolution of legal and illegal drug use in the United States [2,3] and other developed countries [4]. It is also necessary to have these statistics for Latin America analyzing use of these substances and changes over time. To do this, you may consider that in cross-sectional designs the number of participants is important, as well as the number of people invited to participate, which is especially important when you want to determine prevalence [5,6]. This participation proportion has not been presented in Hynes’ study [1].

Therefore, we suggest the authors present the participation proportion of each of the surveys to assess the internal validity of the study, and thus evaluate the potential selection bias in relation to the population not involved in the study (*i.e.*, those who did not participate). We would also recommend presenting weighted and unweighted estimates [6].

## Conclusions

We suggest reporting the participation proportion for each survey and to present weighted and unweighted estimates.
